# Modelling the Tox21 10 K chemical profiles for *in vivo* toxicity prediction and mechanism characterization

**DOI:** 10.1038/ncomms10425

**Published:** 2016-01-26

**Authors:** Ruili Huang, Menghang Xia, Srilatha Sakamuru, Jinghua Zhao, Sampada A. Shahane, Matias Attene-Ramos, Tongan Zhao, Christopher P. Austin, Anton Simeonov

**Affiliations:** 1 Division of Pre-clinical Innovation, National Center for Advancing Translational Sciences, National Institutes of Health, 9800 Medical Center Drive, Rockville, Maryland 20850, USA

## Abstract

Target-specific, mechanism-oriented *in vitro* assays post a promising alternative to traditional animal toxicology studies. Here we report the first comprehensive analysis of the Tox21 effort, a large-scale *in vitro* toxicity screening of chemicals. We test ∼10,000 chemicals in triplicates at 15 concentrations against a panel of nuclear receptor and stress response pathway assays, producing more than 50 million data points. Compound clustering by structure similarity and activity profile similarity across the assays reveals structure–activity relationships that are useful for the generation of mechanistic hypotheses. We apply structural information and activity data to build predictive models for 72 *in vivo* toxicity end points using a cluster-based approach. Models based on *in vitro* assay data perform better in predicting human toxicity end points than animal toxicity, while a combination of structural and activity data results in better models than using structure or activity data alone. Our results suggest that *in vitro* activity profiles can be applied as signatures of compound mechanism of toxicity and used in prioritization for more in-depth toxicological testing.

## Abstract (web summary)

Large-scale *in vitro* assays may reduce the number of toxicological tests carried out in animals. Here, Huang *et al*. report a large dataset containing results of *in vitro* tests of approximately 10,000 chemicals, and use these data to create models that can potentially predict toxicity in humans.

## Graphical abstract

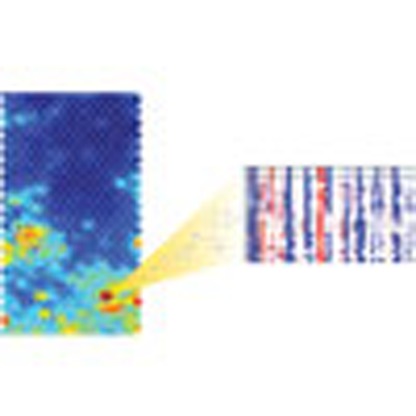

Thousands of chemicals to which humans are exposed have inadequate data on which to predict their potential for toxicological effects. Traditional toxicity testing conducted *in vivo* using animal models provides chemical safety reference to humans, but these methods are expensive and low throughput, and it is often difficult to extrapolate the test results to human health effect because of species differences. High-throughput screening (HTS) techniques are now routinely used in conjunction with computational methods and information technology to probe how chemicals interact with biological systems, both *in vitro* and *in vivo*. Progress is being made in recognizing the patterns of response in genes and pathways induced by certain chemicals or chemical classes that might be predictive of adverse health outcomes in humans. However, as with any new technology, both the reliability and the relevance of the approach need to be demonstrated in the context of current knowledge and practice.

The Tox21 programme^1^,^2^,^3^,^4^, a collaboration between the National Institute of Environmental Health Sciences/National Toxicology Program, the US Environmental Protection Agency/National Center for Computational Toxicology, the National Institutes of Health Chemical Genomics Center (now within the National Center for Advancing Translational Sciences) and the US Food and Drug Administration, aims to identify chemical structure–activity signatures derived through *in vitro* testing that could act as predictive surrogates for *in vivo* toxicity. During the production phase of the Tox21 programme, the Tox21 10 K compound library has been screened against 30 cell-based assays, including nuclear receptors^5^ and stress response pathways^6^, in a quantitative HTS (qHTS) format in triplicate^7^,^8^,^9^,^10^.

Here we review the performance of these assays and the data quality, summarize the activities observed from these assays and evaluate the utility of the data towards achieving the Tox21 goals. We find the *in vitro* assay activity profiles useful for hypotheses generation on compound mechanism of toxicity. These data can be applied, together with chemical structure information, to build predictive models for *in vivo* toxicity and prioritize chemicals for more advanced toxicological tests.

## Results

### Assay performance and activity distribution summary

**Figure 1:**
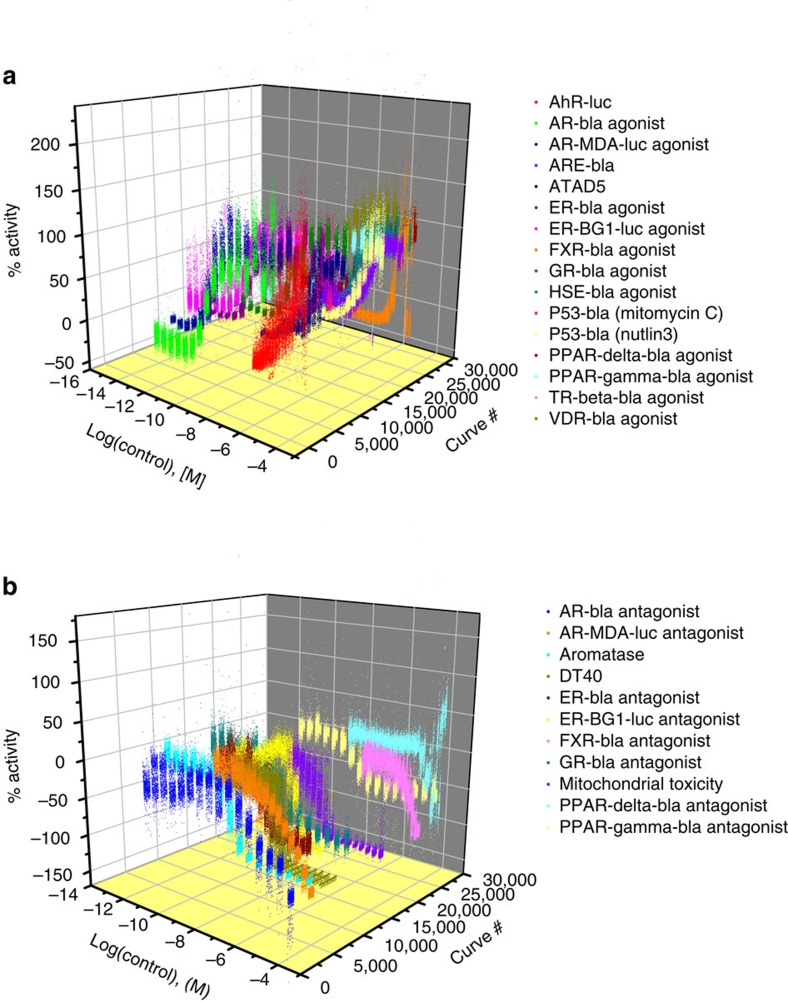
Concentration response data of the positive control compounds for the 30 Tox21 phase II assays. (**a**) Agonist mode assays; (**b**) antagonist mode assays. The positive control compound is plated as 16-pt. titrations in duplicate in the control columns of every assay plate. In the figure, each concentration response curve is from one plate with a total of 408 plates per assay. The consistency of the control response curves is an indicator of good assay performance.

Twelve of the thirty assays screened performed well in the qHTS format with performance statistics^11^ including signal-to-background ratios ≥3-fold, coefficient of variances ≤10% and Z' factors ≥0.5 (Table 1). The other 18 assays, for example, the AR-bla antagonist mode assay, with poorer performance in one or two metrics, for example, lower signal-to-background ratio (<3), were compensated by better performance in other metrics, for example, extremely small coefficient of variance (<5%), such that the overall assay performance still withheld as measured by data reproducibility as described below. The positive control titrations embedded in every plate replicated well across the entire screen (Fig. 1) with variations in AC_50_s <3-fold for 89% of the assays and <4-fold for all assays (Table 1). A more direct measure of assay performance is data reproducibility. Reproducibility^9^ as represented by active match, inactive match, inconclusive and mismatch rates was calculated for all assays (Table 2) screened against the three copies of the 10 K library with compounds plated in different well locations in each copy. Seventeen of the thirty assays scored (score=2 × %active match+%inactive match – %inconclusive - 2 × %mismatch) >90 (grade A) in terms of reproducibility with <0.5% mismatches in activity (Table 2). Eleven assays had reproducibility scores between 80 and 90 (grade B) with mismatch rates <1%. Only two assays, the wild-type DT40 and the GR-bla antagonist mode assay, scored below 80, but still above 75, with 1–2% mismatch rates. For the same sample, the average AC_50_ differences between the three runs were <2-fold for all the assays (Table 2).

**Figure 2:**
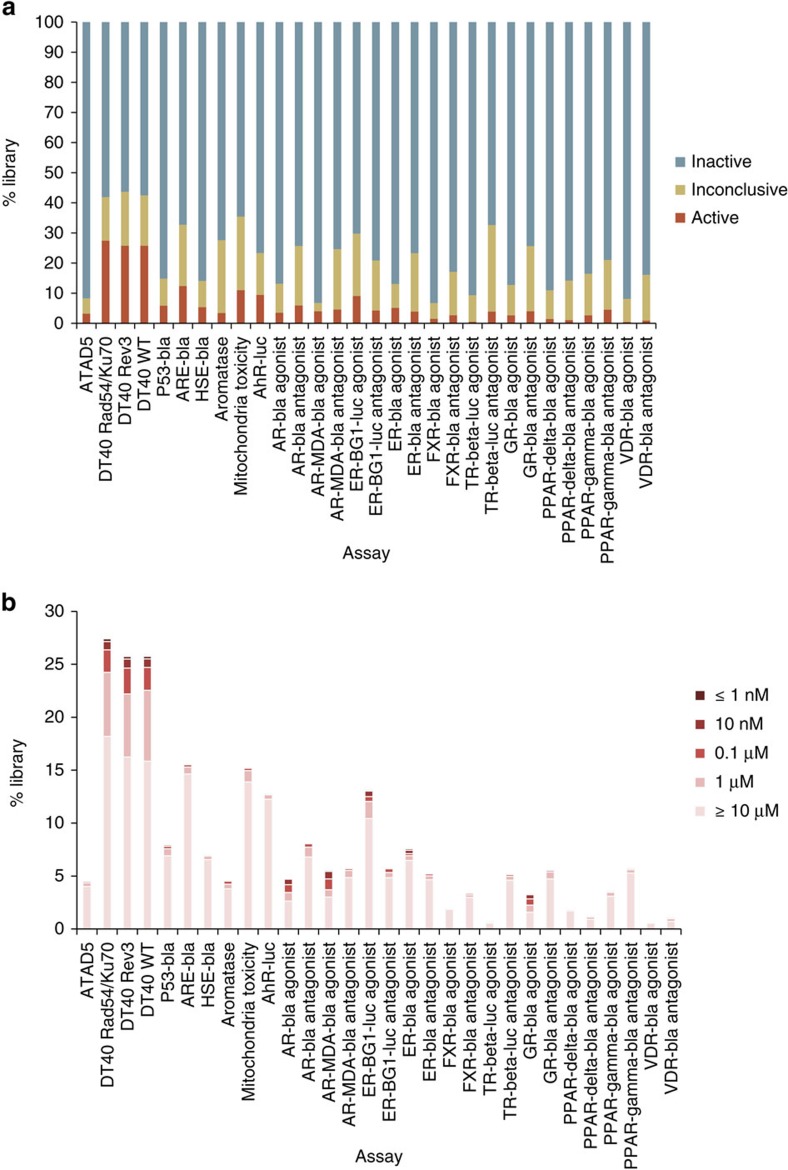
Activity distribution of the Tox21 10 K library screened against the 30 assays. (**a**) Activity outcome distribution; (**b**) potency distribution.

The 30 assays screened against the Tox21 10 K collection showed a wide spectrum of activities (Fig. 2) with active rates ranging from 0.43% (VDR-bla agonist mode assay) to 27.4% (DT40 *Rad54/Ku70* mutant assay; Fig. 2a) and potencies ranging from subnanomolar to tens of micromolar (Fig. 2b). The average active rate of the 30 assays was 6.5%. The AR-MDA-luc agonist mode assay had the largest fraction of potent compounds (33.3% of actives had AC_50_ <1 μM), whereas the FXR-bla agonist mode assay had no active compound with AC_50_ <1 μM.

### Clustering compounds by activity profile

**Figure 3:**
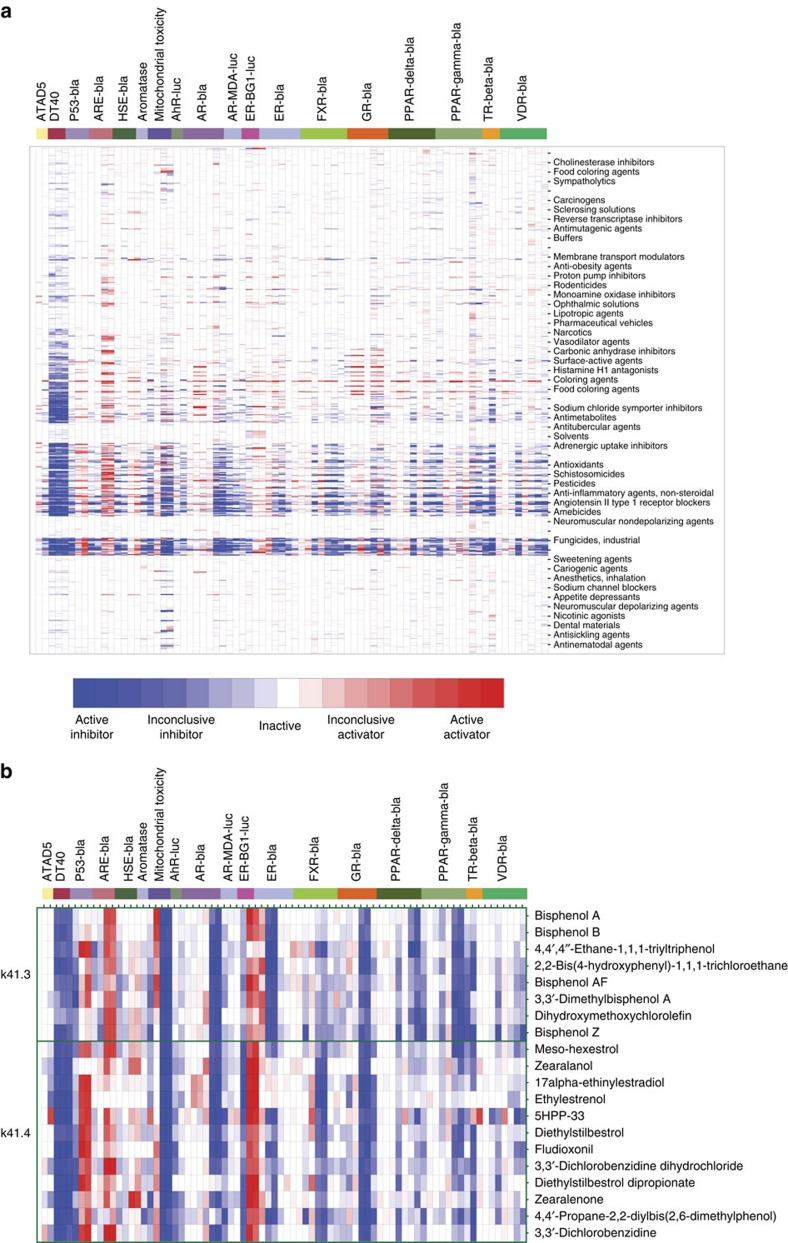
Clustered activity profiles of the Tox21 10 K library. In the heat map, each row is a compound and each column is an assay readout. The heat map is coloured by the compound activity outcome, such that darker red or blue colours indicate more confident activators (red) or inhibitors (blue). Compounds are grouped into clusters of similar activity profiles. Each cluster of compounds is labelled by the most significantly enriched MeSH PA term in that cluster measured by a Fisher's exact test. (**a**) All 10 K compounds; (**b**) example clusters of oestrogenic compounds.

The 10 K compounds were grouped into 610 clusters by their activity profile similarity and each cluster was examined for enriched Medical Subject Headings (MeSH; http://www.ncbi.nlm.nih.gov/mesh) pharmacological action (PA) terms (see Online Methods for details). Figure 3 shows the clustered activity profiles and the most significantly enriched MeSH PA term in each cluster (Supplementary Data 1). Of the 553 clusters that contain at least one compound with known MeSH PA (Supplementary Data 1), 544 clusters have significantly enriched terms with *P*<0.05 (Fisher's exact test), 362 clusters with *P*<0.01 (Fisher's exact test) and 89 clusters with *P*<0.001 (Fisher's exact test). This result indicates that compounds with similar activity profiles as determined in the Tox21 screens tend to share similar annotated modes of action (MOAs). For example, all compounds in cluster k36.15 are annotated as cardiotonic agents, including deslanoside, digitoxin, digoxin, ouabain and proscillaridin, which are cardiac glycosides that act by inhibiting Na/K channels^12^. Cluster k30.6 is enriched with statins, including atorvastatin, fluvastatin and cerivastatin, which are hydroxymethylglutaryl-CoA reductase inhibitors^13^. Seven of the eight compounds in cluster k30.9 are antineoplastic antimetabolites, with the only exception of *N*-butyl-*N*′-nitro-*N*-nitrosoguanidine, which does not have a MeSH PA annotation but is a known DNA alkylating agent^14^. Another example is cluster k41.4, which is enriched with oestrogenic compounds, such as 17alpha-ethinylestradiol and non-steroidal oestrogens such as diethylstilbestrol and zearalenone (Fig. 3b). A neighbouring cluster, k41.3, contains bisphenol type compounds such as bisphenols A, B, Z and AF. Even though most of the bisphenols do not have a MeSH PA assigned, they are known oestrogenic compounds, as well^15^. These compounds are not only structurally similar but also share similar activity profiles (Fig. 3b).

Moreover, 537 clusters contain both compounds with known MeSH PA and compounds with no MeSH PA annotation available. If most compounds in the same cluster share similar annotated MOA, then we can use this information to hypothesize on the MOA of the unknown compounds. For example, the pesticide fludioxonil does not have a MeSH PA assigned but co-clustered with the oestrogenic compounds in k41.4, consistent with recent reports on its endocrine disrupting activity^16^. Similarly, the MeSH term ‘anti-inflammatory agents' is significantly enriched in cluster k22.1 (Fisher's exact test: *P*=4.90 × 10^−12^). Most of the compounds in this cluster are glucocorticoids with similar steroid type structure. Six out of the sixteen compounds in this cluster do not have MeSH PA annotations, including metformin, a diabetes drug with a distinct structure. Metformin decreases hyperglycemia primarily by suppressing glucose production in the liver (hepatic gluconeogenesis)^17^; however, the molecular target of metformin is not clearly understood. The present co-clustering of metformin with the anti-inflammatory glucocorticoids indicates that it may act in a manner similar to that of the glucocorticoids. Metformin was identified as an active agonist in both the glucocorticoid receptor (GR) and androgen receptor (AR) assays. Ampiroxicam is another non-steroidal drug found in k22.1 without a MeSH PA annotation. A literature search on ampiroxicam revealed that it is also an anti-inflammatory drug^18^ supporting the utility of clustering by activity profile as an indicator for compound MOA.

### Modelling *in vivo* toxicity with *in vitro* and structural data

**Figure 4:**
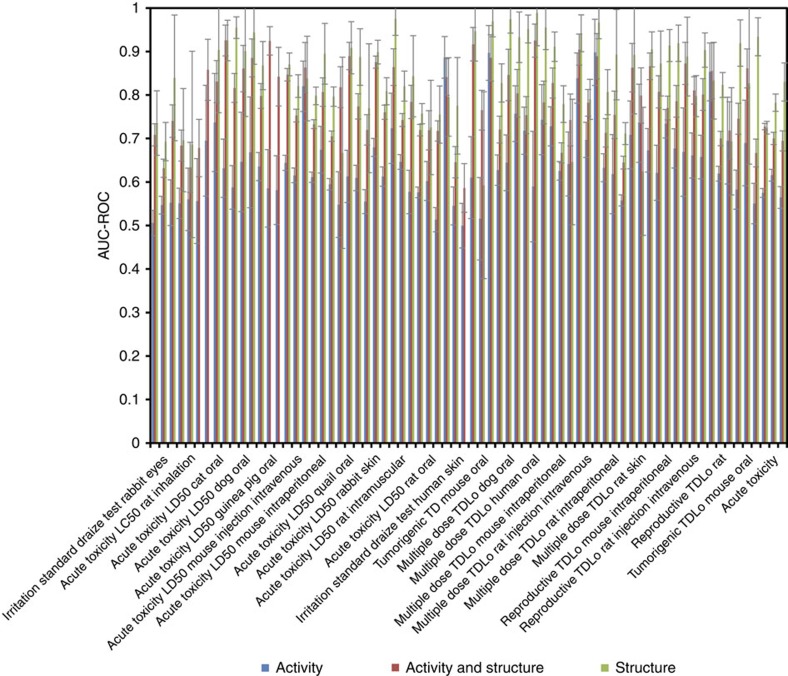
AUC-ROC values of predictive models built for 72 *in vivo* toxicity end points using the Tox21 10 K *in vitro* assay activity data, compound structure data and a combination of the assay and structure data. Columns of AUC-ROC values are shown as mean±s.d. (*n*=100).

For benchmarking our models against available *in vivo* data, we used 72 end points derived from the Registry of Toxic Effects of Chemical Substances database as described in the Methods section. These models are based on compound assay activity profiles clusters (activity-based models) or structure clusters (structure-based models) or both (combined models). The detailed model construction process is described in the Online Methods. The premise for these models is that compounds that share similar *in vitro* signatures and/or structure features are likely to show similar *in vivo* effects. We first attempted to build models for each of the 72 end points using the Tox21 phase II assay activity profiles. The average area under the receiver operating characteristic (ROC) curve (AUC-ROC) values from the 100 randomizations of each model are shown in Fig. 4 and listed in Supplementary Table 1. The AUC-ROC values for the 72 toxicity end points ranged from 0.50 (rat multiple dose inhalation) to 0.90 (rat multiple dose intramuscular injection) with an average of 0.64. In all, 7 of the 72 end points had good predictive models with average AUC-ROC values >0.75. There are five human toxicity end points, including standard Draize test for human skin irritation, multiple dose toxicity data (TDLo) through oral exposure from human females, human males and humans (gender not specified), and reproductive toxicity data (TDLo) through oral exposure from human females. The models built for these end points performed significantly better than the models of the 42 mouse/rat toxicity end points and the 7 rabbit toxicity end points comparing the AUC-ROC values (*t*-test: *P*<0.05). The average AUC-ROC values were 0.75 for the human toxicity models, 0.65 for the mouse/rat models and 0.59 for the rabbit toxicity models.

The compound structure-based models showed overall better performance than the activity-based models, underlying the ongoing need to further expand the battery of *in vitro* assays. The AUC-ROC values for the 72 toxicity end points ranged from 0.59 (rat multiple dose inhalation) to 0.93 (acute toxicity in dog) with an average of 0.78 and 45 of the 72 end points had good predictive models with average AUC-ROC values >0.75 (Fig. 4 and Supplementary Table 1). However, the models built for toxicity end points from different species did not show any significant difference in their predictive performance. Compared with the activity-based models, the performance improved significantly for the mouse/rat and rabbit toxicity models with average AUC-ROC values increased to 0.77 and 0.76, respectively, but not as significantly for the human toxicity models (average AUC-ROC=0.81).

We then attempted to combine the compound structure and assay data in an effort to further improve the models. Models were built for only 67 of the 72 toxicity end points because the more stringent criteria (requiring compounds to co-cluster by both structure and activity) used to form the consensus clusters, which were the basis for the combined models, resulted in smaller clusters such that the compounds with data on the remaining 5 end points all became singletons when split into training and test sets. Nevertheless, the combined models built for the 67 end points showed significantly better performance than the structure-based models with an average AUC-ROC of 0.84 (compared to the average AUC-ROC of 0.78 for the structure-based models; *t*-test: *P*=1.7 × 10^−7^), and 55 out of the 67 end points achieved AUC-ROC values >0.75 (Fig. 4 and Supplementary Table 1). Similar to the structure-based models, the species difference between the model performances disappeared. The animal toxicity models showed a larger improvement in model performance than the human toxicity models, for example, the average AUC-ROC values for the mouse/rat models increased from 0.77 to 0.84 (*t*-test: *P*=3.7 × 10^−6^), whereas the average performance of the human models increased from 0.81 to 0.86 (*P*>0.05).

## Discussion

The Tox21 10 K collection has been screened against 30 assays, yielding high-quality data sets with reproducibility scores >85. In this study, we summarized the activities observed from these assays. Further analyses of the data are currently underway to assess the biological relevance of the assay results, that is, whether the actives identified by an assay are truly perturbing the pathway that is purportedly being measured by the assay and not being results of assay artefacts^19^. For this purpose, sets of reference or tool compounds with known activity in these pathways need to be collected to obtain an estimate of the false positive/negative rates of each assay^8^,^9^,^10^. With our current active identification methods^9^, we found that the compound activity profiles or signatures generated across the 30 assays are useful for MOA hypotheses generation and chemical prioritization^20^. Compounds with unknown MOA that share similar profiles with compounds with known MOA could be prioritized and tested for that hypothesized MOA.

We tested the applicability of the assay data to building predictive models for *in vivo* toxicity end points in comparison with chemical structure data. The predictive performances of most of these models are reasonable but not ideal, and are end point dependent. Our results show that with the current set of assay data chemical structures appear to be more predictive than assay activity profiles for most *in vivo* toxicity end points. One reason for this could be that the assays we have screened so far only focused on two major areas: nuclear receptor signalling and stress response pathways. Although these pathways are important for toxicity, they are far from encompassing all aspects of biology involved in toxic response. In the continuation of the Tox21 programme, more assays will be included to cover additional pathways and targets that could be relevant for toxicity. Moreover, we have observed that not all assays contribute equally to the predictive power of the models, suggesting that it is important to select the relevant assays and to ensure comprehensive coverage. We checked the predictive capacity of each assay of each *in vivo* toxicity end point, and for each end point only a few assays were predictive with AUC-ROC >0.7 (Supplementary Table 1). Assays that measure cell viability took up over half of the most predictive assays.

Species difference is another important contributing factor to the less-than-ideal performance of the models based on assay data. All of the screening data we used in this analysis are derived from cell-based assays using human cells or cell lines, whereas most of the *in vivo* data we are trying to model are collected from animals. According to a 2004 Food and Drug Administration report, 92% of new drugs that passed animal testing failed in human clinical trials because of lack of effect or unexpected toxicity 21. More recent studies show that animal data predicted human outcomes only around half of the time^22^. It is thus not surprising that human *in vitro* cell line data did not show high-predictive power when applied to predict animal toxicity data. To better assess the predictive value of the human *in vitro* assay data, *in vivo* human toxicity data, that is, clinical toxicity data presently not readily available to the public, are required.

Comparison of the models for the few human toxicity end points with the models for animal toxicity showed that the assay data-based models performed markedly better in predicting human toxicity than animal toxicity; in contrast, the structure-based models did not show this species selectivity. Consistent with our findings, a previous small-scale study testing 50 compounds reported similar observations that acute human systemic toxicity was predicted better by human cell lines than animal cell lines^23^. Furthermore, differences in experimental conditions between *in vitro* and *in vivo* studies, such as dosage, timing and metabolic capacity differences, could affect the extrapolation from *in vitro* to *in vivo* results. Under qHTS conditions, most compounds are tested in a fixed concentration range up to 100 μM, and all of the assays used are short-term assays with compound exposure time ranging from a few hours to a day, whereas for *in vivo* studies compounds are tested at much wider dose ranges and time spans up to months and years^24^.

Encouragingly, combining structure and activity information significantly improved the model performance for most of the *in vivo* end points. This phenomenon has been observed previously and reviewed recently^25^. Our results further corroborate the value of the *in vitro* assay data when applied to *in vivo* toxicity prediction. However, the applicability domain of the combined models is limited by the availability of *in vivo* data for a number of toxicity end points. This again highlights the importance of having easy access to more high-quality *in vivo* data.

**Figure 5:**
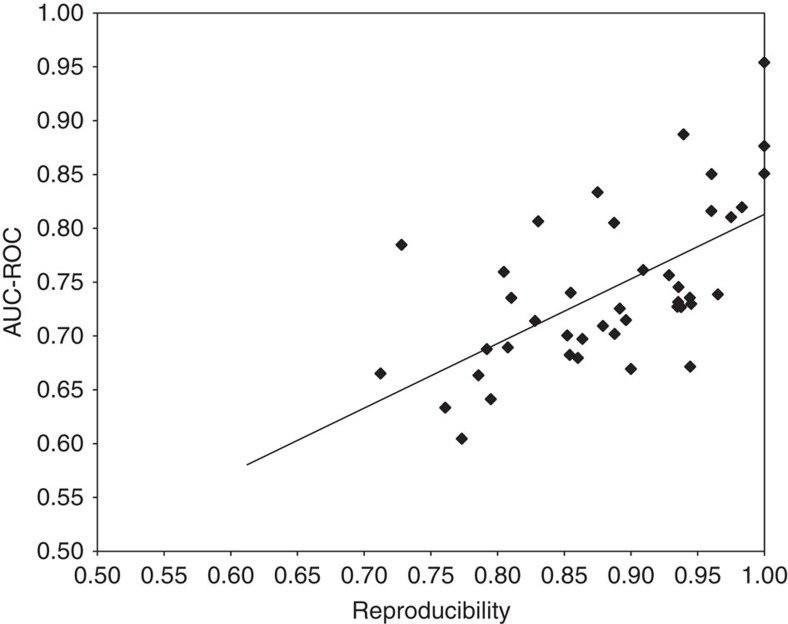
Correlation between reproducibility of *in vivo* data and model performance. A positive correlation (Pearson correlation: *r*=0.61, *P*=1.51 × 10^−5^) is found between the reproducibility of the compounds tested in replicates and the model performance (AUC-ROC). Models built for more reproducible data showed better predictive power.

Data quality is another important factor affecting model performance—a prediction could only be as good as the data it is based on. All measurements have errors or variations associated. In this study, we evaluated the quality of the *in vitro* qHTS data in terms of reproducibility (Table 2). We also checked the reproducibility of the *in vivo* data for which we tried to model following a similar approach using compounds with replicate measurements in each *in vivo* toxicity end point. We found that for those end points with at least 20 compounds that had replicates, there was a significant correlation (Pearson correlation: *r*=0.61, *P*=1.51 × 10^−5^) between the reproducibility of the replicates and the performance (AUC-ROC) of the model built for that end point, such that models built for more reproducible data showed better predictive power (Fig. 5). This observation suggests that improving data quality would help further improve the performance of *in vivo* toxicity prediction models. The high-quality winning models resulting from the recent Tox21 Data Challenge (https://tripod.nih.gov/tox21/challenge), a crowdsourcing effort that asked participants to build predictive models for the *in vitro* assay data based on chemical structure, provide additional evidence for the importance of data quality.

In summary, the Tox21 10 K chemical library has been screened against a panel of nuclear receptor and stress response pathway assays, producing the largest set of high-quality *in vitro* toxicity data known to date. Although data analysis and interpretation are still underway, the compound activity profiles generated from this study have been shown useful for MOA hypotheses generation and chemical prioritization. Here, we built and assessed predictive models for various *in vivo* toxicity end points using *in vitro* qHTS data and compound structure data. The *in vitro* assay data-based models were distinctly better at predicting human toxicity end points than animal toxicity. More human toxicity data and high-quality *in vivo* data are critical in assessing the true predictive power of *in vitro* data-based models of *in vivo* toxicity. Combing structure and activity data resulted in better models than those built with structure or activity data alone reinforcing the value of *in vitro* assay data in toxicity prediction. The scale and high-resolution nature of the data provided within this screening offer the opportunity for researchers worldwide to derive new insights from this valuable resource in a manner akin to previous crowdsourcing efforts (https://tripod.nih.gov/tox21/challenge/)^26^,^27^. We have made publicly available all the HTS results (http://www.ncbi.nlm.nih.gov/pcassay?term=tox21) as well as the clustering results used for modelling and the Tox21 compound library information online (http://tripod.nih.gov/tox/filedownload/).

## Methods

### Tox21 chemical library

The Tox21 10 K library consists of compounds mostly procured from commercial sources by the Environmental Protection Agency (http://www.epa.gov/ncct/dsstox/sdf_tox21s.html), National Toxicology Program and National Institutes of Health Chemical Genomics Center^28^, for a total of greater than 10,000 plated compound solutions consisting of 8,599 unique chemical substances including pesticides, industrial chemicals, food additives and drugs. The main criteria for selection of the Tox21 compounds included, but were not limited to, known or perceived environmental hazards or exposure concerns, physicochemical properties indicating suitability for HTS (molecular weight, volatility, solubility, logP), commercial availability and cost. In addition, the Tox21 Chemical Selection Group designated 88 diverse compounds in the Tox21 library to serve as internal controls^9^ to assess assay reproducibility and examine positional plate effects: these were included as duplicates in all screening plates^7^. The structures and annotations of the Tox21 10 K library have been deposited into PubChem (http://www.ncbi.nlm.nih.gov/pcsubstance/?term=tox21).

### Assays and qHTS data analysis

Two areas were the initial focus in the Tox21 phase II screening including nine nuclear receptor targets and seven stress response pathways, selected based on their biological and toxicological relevance, public interest and adaptability to miniaturization and automated screening. The assays were run in different modes (agonist versus antagonist) and/or formats (full length versus partial receptor) as detailed below, totaling 30 assays. Two reporter gene systems, β-lactamase (bla) and luciferase (luc), were used in this study^5^. All cell-based assays were multiplexed with a cell viability assay in the same assay well. Although bla-based assays were multiplexed with a luminescence-based cell viability assay (CellTiter-Glo viability assay, Promega), luc-based assays were multiplexed with a fluorescence-based cell viability assay (Cell Titer-Fluor viability assay, Promega). The nuclear receptor assays, including oestrogen receptor alpha, ligand-binding domain (ER-bla), oestrogen receptor alpha, full length (ER-BG1-luc), androgen receptor, ligand-binding domain (AR-bla), androgen receptor, full length (AR-MDA-luc), glucocorticoid receptor (GR-bla), farnesoid X receptor (FXR-bla), peroxisome proliferator-activated receptor delta (PPAR-delta-bla), peroxisome proliferator-activated receptor gamma (PPAR-gamma-bla), thyroid hormone receptor (TR-Luc), vitamin D receptor (VDR-bla) and aryl hydrocarbon receptor (AhR-luc) assays, were screened in both agonist and antagonist modes. Aromatase^29^,^30^, mitochondrial toxicity^31^ and DNA repair deficient isogenic chicken DT40 cell viability (DT40) assays^32^ were screened in antagonist mode. A number of stress response pathway assays, including ATAD5-luc^33^, a genotoxicity assay, p53-bla, antioxidant responsive element (ARE-bla)^34^ and heat-shock factor response element (HSE-bla) assays, were screened in agonist mode. Detailed assay protocols can be found in PubChem (http://www.ncbi.nlm.nih.gov/pcassay?term=tox21).

In primary screening, all compounds were tested as three independent runs, with each of the three instances of a compound sample residing in a different location on a different compound plate across replicates. Each replicate set of plates was tested on a different day using a different batch of cells. Analysis of compound concentration–response data was performed as previously described^5^. Briefly, raw plate reads for each titration point were first normalized relative to the positive control compound (agonist mode: 100%; antagonist mode: 0%) and dimethylsulphoxide (DMSO)-only wells (agonist mode: 0%; antagonist mode: -100%) as follows: % Activity=((*V*_compound_ – *V*_DMSO_)/(*V*_pos_ – *V*_DMSO_)) × 100, where *V*_compound_ denotes the compound well values, *V*_pos_ denotes the median value of the positive control wells and *V*_DMSO_ denotes the median values of the DMSO-only wells, and then corrected by applying an in-house pattern correction algorithm using compound-free control plates (that is,, DMSO-only plates) at the beginning and end of the compound plate stack. Concentration–response titration points for each compound were fitted to a four-parameter Hill equation yielding concentrations of half-maximal activity (AC_50_) and maximal response (efficacy) values. Compounds were designated as Class 1–4 according to the type of concentration–response curve observed^5^,^35^. Curve classes are heuristic measures of data confidence, classifying concentration–responses on the basis of efficacy, the number of data points observed above background activity, and the quality of fit. Each curve class was then converted to a curve rank as previously described^5^ such that more potent and efficacious compounds with higher quality curves were assigned a higher rank. Curve ranks should be viewed as qualitative descriptors of the concentration response profile of the compound. Compound reproducibility was assessed by calculating the reproducibility of the curve ranks of each compound generated from the triplicate runs^9^. A reproducibility score was calculated for each assay using the formula: score=2 × %active match+%inactive match-2 × %mismatch-%inconclusive^5^.

### Data sources

All Tox21 phase II qHTS data are available in PubChem (http://www.ncbi.nlm.nih.gov/pcassay?term=tox21; see Accession Codes section for assay IDs). *In vivo* toxicity data were retrieved from the Registry of Toxic Effects of Chemical Substances database compiled by Leadscope (Leadscope, Inc.). This compilation contains 129 different toxicity end points including acute toxicity, hepatotoxicity, reproductive toxicity, carcinogenicity and skin and eye irritation from various species such as human, rodents, primates and birds on >10,000 molecules, 6,447 of which overlap with compounds in the Tox21 10 K library. Most of these compounds do not have data available for every toxicity end point. Only the end points that have at least 50 active/toxic calls and 50 inactive/non-toxic calls were kept for further analysis. A total of 68 of the 129 end points met this data availability requirement. In addition, we created three composite end points by aggregating the acute toxicity, reproductive toxicity and the tumorigenic end points, respectively, for a total of 72 end points. For compounds with LD_50_ data available, an LD_50_ of <300 mg kg^−1^ was considered toxic^36^. For other end points, compounds with toxicity measures falling into the top 35 percentile were considered toxic. For composite end points, compounds that are toxic in more than half of the component end points were considered toxic for the composite call. The 72 selected end points and the number of toxic/non-toxic compounds in each end point are listed in Supplementary Table 1. MeSH (http://www.ncbi.nlm.nih.gov/mesh) PA terms were used for compound MOA annotations.

### Clustering and modelling for *in vivo* toxicity

The 10 K library was clustered based on similarity in its members' activity profiles (measured by curve rank) across the 30 assays using the self-organizing map (SOM) algorithm^37^, resulting in 610 clusters. Each cluster was evaluated for enrichment of 363 MeSH PA terms using the Fisher's exact test. The 10 K library was also clustered with the SOM algorithm based on structural similarity using the Leadscope (Leadscope, Inc.) structure fingerprints resulting in 999 clusters. The SOM algorithm clustered the compound activity profiles or structure fingerprints based on the similarity between the profiles measured by pair-wise Euclidean distance, and the analysis was performed using the SOM Toolbox (http://www.cis.hut.fi/projects/somtoolbox/) where detailed documentation of the algorithm can be found. Briefly, the SOM was trained and optimized through 14 phases with 38,000 steps in each phase to minimize the distances between the central data vectors and the compound profiles to form the clusters. The initial learning rate alpha was set to 0.05, which decreased linearly to zero during training. The initial radius of the training area was set to 20 and decreased linearly to one during training. Models were built for the 72 *in vivo* toxicity end points using either the structure (structure-based models) or assay activity (activity-based models) SOM clusters or both. To build models using both the structure and activity SOM clusters, each compound was reassigned to a ‘consensus cluster' such that only compounds that belong to the same structure cluster and the same activity cluster were assigned to the same ‘consensus cluster'. The consensus clusters were used to build the structure–activity combined models. For each model, compounds were randomly split into two groups of approximately equal sizes, one used for training and the other for testing. The randomization was conducted 100 times to generate 100 different training and test sets to evaluate the robustness of the models. For each SOM cluster containing the training compounds, the enrichment of toxic training compounds was determined by a Fisher's exact test. The –log *P*-value from the Fisher's exact test was used as a measure of the toxic potential (toxicity score) of the compounds in this cluster, and evaluated as a predictor of toxicity for test compounds that fall into the same cluster. More significant *P*-values (larger –log *P*-values) indicate a larger probability of toxicity. If a cluster was deficient of toxic compounds, that is, the fraction of toxic compounds in the cluster was smaller than the fraction of toxic compounds in the whole library, the log *P*-value was used instead. Here we denote the toxicity scores obtained from the activity SOM as *p*-activity, those from the structure SOM as *p*-structure, and those using both the activity and structure SOMs as *p*-both. To test model performance, the corresponding SOM cluster or consensus cluster was located for each test set compound and *p*-activity, *p*-structure or *p*-both obtained from the training set was retrieved and compared with the true toxicity outcome of the test compound to determine whether the test compound should be counted as a true positive (TP: toxic and score>cutoff), false positive (FP: non-toxic and score>cutoff), true negative (TN: non-toxic and score ≤cutoff) or false negative (FN: toxic and score ≤cutoff). Model performance was assessed by calculating the AUC-ROC, which is a plot of sensitivity [TP/(TP+FN)] versus (1-specificity [TN/(TN+FP)])^38^. A perfect model would have an AUC-ROC of 1 and an AUC-ROC of 0.5 indicates a random classifier. The random data split and model training and testing were repeated 100 times, and the average AUC-ROC values were calculated for each model.

## Additional information

**Accession codes:** All Tox21 phase II qHTS data have been deposited in PubChem under the following assay IDs: ATAD5: 651632, 720516, 651634; DT40 *Rad54/Ku*: 70743015; DT40 *Rev3*: 743014; DT40 WT: 743015; P53-bla: 651631, 720552, 651633; ARE-bla: 743202, 743219, 743203; HSE-bla: 743210, 743228, 743209; aromatase: 743083, 743139, 743084; mitochondria toxicity: 720635, 720637, 720634; AhR-luc: 743085, 743122, 743086; AR-bla agonist: 743036, 743053; AR-bla antagonist: 743035, 743063, 743033; AR-MDA-luc agonist: 743040; AR-MDA-luc antagonist: 743042, 743054, 743041; ER-BG1-luc agonist: 743079; ER-BG1-luc antagonist: 743080, 743091, 743081; ER-bla agonist: 743075, 743077; ER-bla antagonist: 743069, 743078, 743074; FXR-bla agonist: 743220, 743239, 743218; FXR-bla antagonist: 743217, 743240, 743221; GR-bla agonist: 720691, 720719; GR-bla antagonist: 720692, 720725, 720693; PPAR-delta-bla agonist: 743212, 743227, 743211; PPAR-delta-bla antagonist: 743215, 743226, 743213; PPAR-gamma-bla agonist: 743094, 743140; PPAR-gamma-bla antagonist: 743191, 743199, 743194; TR-beta-luc agonist: 743066; TR-beta-luc antagonist: 743065, 743067, 743064; VDR-bla agonist: 743222, 743241, 743224; VDR-bla antagonist: 743223, 743242, 743225.

**How to cite this article:** Huang, R. *et al*. Modelling the Tox21 10 K chemical profiles for *in vivo* toxicity prediction and mechanism characterization. *Nat. Commun.* 7:10425 doi: 10.1038/ncomms10425 (2016).

## Supplementary Material

Supplementary Table Supplementary Table 1

Supplementary Data 1 Compounds clustered by assay activity profiles and their MeSH pharmacological action (PA) term annotations.

## Figures and Tables

**Table 1:** Tox21 10 K qHTS assay summary statistics*.

Assay	S/B	Z' factor	CV	Positive control	Control AC_50_	Control AC_50_ fold change
AhR-luc	8±4	0.3±0.2	16±15	Omeprazole	49.5 μM	3.14
AR-bla agonist	1.9±0.2	0.2±0.1	5±1	R1881	1.21 nM	2.36
AR-bla antagonist	2.5±0.3	0.7±0.2	4±1	Cyproterone acetate	4.68 μM	2.51
ARE-bla	2.1±0.3	0.70±0.06	5±2	β-Naphthoflavone	1.95 μM	1.29
AR-MDA-luc agonist	6.6±0.6	0.68±0.06	15±2	R1881	14.3 pM	1.52
AR-MDA-luc antagonist	17±10	0.67±0.07	8±2	Nilutamide	15.3 μM	1.49
Aromatase	6.2±0.8	0.80±0.03	4.7±0.9	Letrozole	6.70 nM	1.32
DT40 *Rad54/Ku70*	40±4	0.8±0.2	6±2	Tetra-octyl ammonium bromide	416 nM	1.63
DT40 WT	40±7	0.79±0.08	7±3	Tetra-octyl ammonium bromide	594 nM	1.79
DT40 *Rev3*	40±9	0.79±0.09	6±2	Tetra-octyl ammonium bromide	440 nM	1.5
ATAD5	6.0±0.9	0.73±0.04	14±2	5-Fluorouridine	2.12 μM	2.11
ER-bla agonist	4.7±0.6	0.53±0.09	4±2	β-Estradiol	332 pM	1.51
ER-bla antagonist	3.3±0.8	0.4±0.1	11±3	4-Hydroxy tamoxifen	5.13 nM	1.58
ER-BG1-luc agonist	2.5±0.3	0.5±0.2	10±5	β-Estradiol	29.0 pM	3.75
ER-BG1-luc antagonist	8.0±0.9	0.77±0.07	6±2	4-Hydroxy tamoxifen	73.2 nM	1.28
FXR-bla agonist	4.0±0.7	0.3±0.2	7±1	Chenodeoxycholic acid	29.8 μM	1.29
FXR-bla antagonist	4.4±0.9	0.67±0.09	3±1	Guggulsterone	36.7 μM	1.3
TR-beta-luc agonist	9±2	0.63±0.07	12±3	T3	41.9 pM	2.14
TR-beta-luc antagonist	5.0±0.9	0.7±0.1	6±3	NA	NA	NA
GR-bla agonist	3.0±0.2	0.73±0.05	3.3±0.8	Dexamethasone	3.64 nM	1.36
GR-bla antagonist	1.9±0.1	0.5±0.1	4.8±0.9	Mifeprostone	1.71 nM	2.16
HSE-bla	3.9±0.5	0.46±0.08	4±1	17-AAG	45.3 nM	1.7
Mitochondria toxicity	6±3	0.6±0.2	8±3	FCCP	96.2 nM	2.79
P53-bla	3.1±0.4	0.6±0.2	6±2	Mitomycin C	1.53 μM	1.62
PPAR-delta-bla agonist	2.5±0.3	0.67±0.06	6±1	L-165,041	36.4 nM	1.8
PPAR-delta-bla antagonist	2.2±0.2	0.57±0.06	4.3±0.6	MK886	38.5 μM	1.95
PPAR-gamma-bla agonist	2.4±0.1	0.73±0.04	5±2	Rosiglitazone	11.8 nM	1.85
PPAR-gamma-bla antagonist	2.1±0.2	0.6±0.3	5±2	GW9662	2.47 nM	3.3
VDR-bla agonist	1.9±0.2	0.5±0.1	5±1	1α, 25-Dihydroxy vitamin D3	35.0 pM	1.97
VDR-bla antagonist	2.7±0.2	0.55±0.06	7±1	NA	NA	NA

AC_50_, concentration at 50% activity, AC_50_ fold change=10^SD(log AC50)^; CV, coefficient of Variance (derived from the negative control wells); NA, not applicable; qHTS, quantitative high-throughput screening; S/B, signal to background.

^*^Data are derived from the positive and negative control wells on each plate and presented as mean±standard deviation (*n*=408)

**Table 2:** Assay performances measured by reproducibility of the Tox21 10 K triplicate runs*.

Assay	Active match (%)	Inactive match (%)	Inconclusive (%)	Mismatch (%)	AC_50_ fold change	Score†
ATAD5	5.34	91.78	2.84	0.05	1.30	99.51
DT40 *Rad54/Ku70*	23.92	59.47	16.50	0.10	1.32	90.61
DT40 *Rev3*	22.38	58.07	19.29	0.26	1.40	83.02
DT40 WT	21.07	58.89	18.65	1.39	1.49	79.58
P53-bla	11.10	84.86	4.04	0.00	1.29	103.02
ARE-bla	15.29	68.34	15.65	0.70	1.76	81.85
HSE-bla	7.57	86.38	6.05	0.00	1.45	95.46
Aromatase	15.94	73.08	10.66	0.30	1.44	93.66
Mitochondria toxicity	17.57	67.52	14.33	0.55	1.53	87.20
AhR-luc	8.86	78.78	12.24	0.10	1.82	84.05
AR-bla agonist	5.36	86.88	7.44	0.30	1.77	89.55
AR-bla antagonist	15.46	75.07	9.38	0.09	1.35	96.44
AR-MDA-luc agonist	4.14	93.65	2.20	0.00	1.36	99.74
AR-MDA-luc antagonist	13.22	76.44	10.07	0.27	1.48	92.28
ER-BG1-luc agonist	16.43	71.22	12.05	0.28	1.52	91.46
ER-BG1-luc antagonist	12.03	79.72	7.96	0.29	1.48	95.25
ER-bla agonist	7.01	87.11	5.87	0.01	1.36	95.25
ER-bla antagonist	9.84	77.86	11.95	0.34	1.49	84.90
FXR-bla agonist	2.46	93.87	3.65	0.02	1.53	95.09
FXR-bla antagonist	7.50	83.04	9.35	0.11	1.73	88.48
GR-bla agonist	6.67	87.49	5.75	0.10	1.37	94.89
GR-bla antagonist	9.73	75.11	13.13	2.01	1.81	77.40
PPAR-delta-bla agonist	3.46	90.79	5.71	0.04	1.71	91.91
PPAR-delta-bla antagonist	5.67	86.73	7.49	0.10	1.73	90.37
PPAR-gamma-bla agonist	8.79	83.87	7.02	0.31	1.61	93.81
PPAR-gamma-bla antagonist	8.61	79.88	11.05	0.44	1.90	85.15
TR-beta-luc agonist	2.13	90.72	7.15	0.00	1.38	87.84
TR-beta-luc antagonist	17.15	68.35	14.18	0.32	1.39	87.82
VDR-bla agonist	2.25	92.53	5.19	0.03	1.62	91.79
VDR-bla antagonist	5.41	86.41	8.11	0.08	1.53	88.97

^*^Active match is the percentage of compounds that were reproducibly active, inactive match is the percentage of compounds that were reproducibly inactive, and mismatch is the percentage of compounds that showed conflicting activities in the triplicate runs. A compound is assigned inconclusive if its activity in the triplicate runs was not clearly active or inactive to make a conclusive reproducibility call. Detailed definitions of the reproducibility calls can be found in our previous report^9^. AC_50_ fold change is the average AC_50_ differences in fold of the active compounds in the triplicate runs.

^†^Score=2 × active match+inactive match – inconclusive - 2 × mismatch.
