# Supplementary material for: Modelling the Tox21 10 K chemical profiles for in vivo toxicity prediction and mechanism characterization
Source: Nat Commun. 2016 Jan 26;7:10425. doi: 10.1038/ncomms10425 (PMC4777217; doi:10.1038/ncomms10425)
Supplement: Supplementary Table — 1 [file ncomms10425-s1.pdf]

**Supplementary Table 1**. Activity distribution and model performance for each in vivo toxicity endpoint

| In vivo endpoint | Toxic | non-Toxic | AUC-ROC (activity model) | AUC-ROC (structure model) | AUC-ROC (combined model) | Most predictive assay | AUC-ROC (most predictive assay) |
|---|---|---|---|---|---|---|---|
| acute toxicity LC50 Mouse Inhalation | 200 | 100 | 0.55±0.05 | 0.74±0.04 | 0.84±0.14 | DT40 WT | 0.62 |
| acute toxicity LC50 Rat Inhalation | 571 | 144 | 0.55±0.03 | 0.68±0.04 | 0.7±0.12 | DT40 Rev3 | 0.62 |
| acute toxicity LC50 Rat Inhalation | 82 | 136 | 0.56±0.07 | 0.63±0.06 | 0.69±0.21 | VDR-BLA antagonist viability | 0.61 |
| acute toxicity LD50 Bird Oral | 197 | 60 | 0.56±0.1 | 0.68±0.07 | N/A | FXR-BLA agonist ch2 | 0.58 |
| acute toxicity LD50 Cat Oral | 82 | 57 | 0.7±0.13 | 0.86±0.07 | N/A | DT40 WT | 0.69 |
| acute toxicity LD50 Chicken Oral | 74 | 106 | 0.74±0.11 | 0.83±0.05 | 0.9±0.14 | AR-MDA antagonist viability | 0.60 |
| acute toxicity LD50 Dog Injection intravenous | 281 | 85 | 0.63±0.07 | 0.93±0.04 | 0.93±0.05 | DT40 Rev3 | 0.72 |
| acute toxicity LD50 Dog Oral | 188 | 348 | 0.59±0.05 | 0.82±0.03 | 0.95±0.02 | TR-beta antagonist viability | 0.58 |
| acute toxicity LD50 Duck Oral | 63 | 105 | 0.65±0.11 | 0.86±0.05 | 0.9±0.15 | ER-BG1 agonist | 0.60 |
| acute toxicity LD50 Guinea pig Intraperitoneal | 107 | 73 | 0.67±0.13 | 0.89±0.04 | 0.94±0.07 | DT40 WT | 0.70 |
| acute toxicity LD50 Guinea pig Oral | 189 | 405 | 0.64±0.03 | 0.8±0.03 | 0.87±0.06 | DT40 Rev3 | 0.67 |
| acute toxicity LD50 Mammal | 76 | 118 | 0.59±0.09 | 0.92±0.03 | N/A | DT40 Rev3 | 0.65 |
| acute toxicity LD50 Mammal Oral | 60 | 186 | 0.58±0.08 | 0.84±0.07 | N/A | DT40 Rad54/Ku70 | 0.67 |
| acute toxicity LD50 Mouse Injection intravenous | 1576 | 562 | 0.64±0.02 | 0.85±0.01 | 0.87±0.03 | DT40 WT | 0.70 |
| acute toxicity LD50 Mouse Injection subcutaneous | 687 | 1070 | 0.62±0.02 | 0.75±0.01 | 0.82±0.03 | DT40 WT | 0.61 |
| acute toxicity LD50 Mouse Intramuscular | 111 | 144 | 0.82±0.06 | 0.86±0.06 | 0.84±0.1 | TR-beta antagonist | 0.75 |
| acute toxicity LD50 Mouse Intraperitoneal | 1618 | 1360 | 0.61±0.01 | 0.73±0.01 | 0.8±0.02 | DT40 WT | 0.64 |
| acute toxicity LD50 Mouse | 150 | 199 | 0.67±0.05 | 0.81±0.03 | 0.89±0.07 | DT40 WT | 0.67 |

| | | | | | | | |
|---|---|---|---|---|---|---|---|
| acute toxicity LD50 Mouse Oral | 847 | 2740 | 0.59±0.01 | 0.71±0.01 | 0.8±0.02 | DT40 Rev3 | 0.61 |
| acute toxicity LD50 Quail Oral | 101 | 137 | 0.55±0.08 | 0.82±0.05 | 0.67±0.22 | ER-BLA agonist ch2 | 0.59 |
| acute toxicity LD50 Rabbit Injection intravenous | 362 | 102 | 0.61±0.06 | 0.89±0.03 | 0.91±0.06 | DT40 Rev3 | 0.74 |
| acute toxicity LD50 Rabbit Oral | 201 | 559 | 0.61±0.03 | 0.77±0.03 | 0.89±0.06 | ARE ch2 | 0.61 |
| acute toxicity LD50 Rabbit Skin | 117 | 992 | 0.55±0.03 | 0.72±0.03 | 0.77±0.15 | DT40 WT | 0.58 |
| acute toxicity LD50 Rat Injection intravenous | 946 | 420 | 0.68±0.02 | 0.88±0.01 | 0.9±0.03 | DT40 Rev3 | 0.73 |
| acute toxicity LD50 Rat Injection subcutaneous | 430 | 923 | 0.61±0.02 | 0.76±0.02 | 0.81±0.03 | DT40 WT | 0.60 |
| acute toxicity LD50 Rat Intramuscular | 87 | 127 | 0.72±0.08 | 0.86±0.04 | 0.97±0.04 | TR-beta antagonist viability | 0.67 |
| acute toxicity LD50 Rat Intraperitoneal | 975 | 1010 | 0.65±0.02 | 0.74±0.01 | 0.82±0.03 | TR-beta antagonist viability | 0.64 |
| acute toxicity LD50 Rat | 129 | 241 | 0.58±0.05 | 0.78±0.04 | 0.84±0.09 | DT40 Rev3 | 0.62 |
| acute toxicity LD50 Rat Oral | 842 | 3390 | 0.58±0.01 | 0.72±0.01 | 0.76±0.02 | TR-beta antagonist viability | 0.60 |
| acute toxicity LD50 Rat Skin | 95 | 532 | 0.6±0.04 | 0.72±0.05 | 0.73±0.11 | ER-BG1 antagonist | 0.56 |
| hepatotoxicity | 926 | 470 | 0.55±0.02 | 0.63±0.02 | 0.69±0.04 | TR-beta agonist | 0.54 |
| irritation standard Draize test Human Skin | 80 | 151 | 0.89±0.05 | 0.84±0.04 | 0.8±0.09 | GR-BLA agonist ratio | 0.75 |
| irritation standard Draize test Rabbit Eyes | 232 | 530 | 0.51±0.03 | 0.71±0.03 | 0.74±0.07 | Aromatase | 0.56 |
| irritation standard Draize test Rabbit Skin | 226 | 535 | 0.51±0.03 | 0.72±0.02 | 0.76±0.07 | P53 ch1 | 0.60 |
| multiple dose TCLo Rat Inhalation micro | 184 | 308 | 0.55±0.04 | 0.65±0.04 | 0.78±0.11 | PPAR-gamma antagonist viability | 0.56 |
| multiple dose TCLo Rat Inhalation | 82 | 154 | 0.5±0.05 | 0.59±0.06 | N/A | P53 ch2 | 0.58 |
| multiple dose TDLo Dog Injection intravenous | 54 | 97 | 0.9±0.06 | 0.89±0.05 | 0.97±0.04 | DT40 WT | 0.76 |
| multiple dose TDLo Dog Oral | 247 | 449 | 0.63±0.03 | 0.72±0.03 | 0.83±0.04 | TR-beta antagonist | 0.62 |
| multiple dose TDLo Human Female Oral | 70 | 133 | 0.64±0.06 | 0.85±0.06 | 0.97±0.03 | AR-BLA antagonist ch2 | 0.64 |

| | | | | | | | |
|---|---|---|---|---|---|---|---|
| multiple dose TDLo Human Male Oral | 73 | 137 | 0.76±0.06 | 0.8±0.05 | 0.93±0.06 | Mitochondria toxicity rhodamine | 0.57 |
| multiple dose TDLo Human Oral | 114 | 215 | 0.72±0.05 | 0.75±0.04 | 0.95±0.03 | ER-BLA agonist ch2 | 0.61 |
| multiple dose TDLo Monkey Oral | 59 | 91 | 0.59±0.13 | 0.93±0.04 | 0.99±0.08 | FXR-BLA agonist viability | 0.62 |
| multiple dose TDLo Mouse Injection subcutaneous | 65 | 132 | 0.74±0.08 | 0.78±0.05 | 0.96±0.05 | AR-BLA antagonist ch1 | 0.59 |
| multiple dose TDLo Mouse Intraperitoneal | 161 | 320 | 0.73±0.05 | 0.83±0.03 | 0.91±0.03 | AR-BLA antagonist ratio | 0.62 |
| multiple dose TDLo Mouse Oral | 411 | 747 | 0.63±0.02 | 0.67±0.02 | 0.78±0.04 | P53 ch1 | 0.60 |
| multiple dose TDLo Rabbit Oral | 97 | 170 | 0.64±0.07 | 0.74±0.06 | 0.65±0.14 | P53 ratio | 0.60 |
| multiple dose TDLo Rat Injection intravenous | 72 | 143 | 0.84±0.06 | 0.9±0.04 | 0.94±0.04 | FXR-BLA antagonist ratio | 0.70 |
| multiple dose TDLo Rat Injection subcutaneous | 175 | 302 | 0.7±0.04 | 0.78±0.03 | 0.79±0.05 | DT40 WT | 0.63 |
| multiple dose TDLo Rat Intramuscular | 56 | 90 | 0.9±0.08 | 0.89±0.05 | 0.97±0.04 | DT40 Rev3 | 0.72 |
| multiple dose TDLo Rat Intraperitoneal | 242 | 473 | 0.63±0.03 | 0.71±0.03 | 0.81±0.05 | DT40 Rev3 | 0.62 |
| multiple dose TDLo Rat | 76 | 148 | 0.62±0.09 | 0.75±0.05 | 0.89±0.1 | P53 ratio | 0.63 |
| multiple dose TDLo Rat Oral | 917 | 1701 | 0.56±0.01 | 0.64±0.01 | 0.71±0.03 | DT40 WT | 0.59 |
| multiple dose TDLo Rat Skin | 61 | 110 | 0.71±0.08 | 0.86±0.06 | 0.89±0.14 | ER-BLA agonist ch2 | 0.65 |
| reproductive TDLo Human Female Oral | 56 | 94 | 0.74±0.09 | 0.8±0.06 | 0.62±0.15 | ER-BLA agonist ch2 | 0.67 |
| reproductive TDLo Mouse Injection subcutaneous | 114 | 216 | 0.67±0.05 | 0.87±0.03 | 0.91±0.04 | DT40 Rev3 | 0.72 |
| reproductive TDLo Mouse Intraperitoneal | 124 | 207 | 0.62±0.06 | 0.81±0.04 | 0.87±0.07 | P53 ratio | 0.67 |
| reproductive TDLo Mouse Oral | 237 | 453 | 0.73±0.03 | 0.77±0.03 | 0.91±0.04 | DT40 Rad54/Ku70 | 0.65 |
| reproductive TDLo Rabbit Oral | 164 | 294 | 0.68±0.05 | 0.79±0.04 | 0.92±0.04 | AR-BLA agonist ratio | 0.63 |
| reproductive TDLo Rat Injection intravenous | 65 | 123 | 0.67±0.1 | 0.87±0.05 | 0.89±0.09 | PPAR-gamma antagonist viability | 0.68 |
| reproductive TDLo Rat Injection subcutaneous | 161 | 294 | 0.66±0.05 | 0.81±0.03 | 0.8±0.05 | ER-BLA agonist ch2 | 0.69 |
| reproductive TDLo Rat Intraperitoneal | 154 | 256 | 0.66±0.05 | 0.8±0.04 | 0.9±0.04 | P53 ratio | 0.66 |

| | | | | | | | |
|---|---|---|---|---|---|---|---|
| reproductive TDLo Rat | 66 | 117 | 0.85±0.07 | 0.86±0.06 | 0.83±0.09 | GR-BLA antagonist ch1 | 0.67 |
| reproductive TDLo Rat Oral | 555 | 1021 | 0.62±0.02 | 0.7±0.02 | 0.82±0.03 | DT40 Rev3 | 0.62 |
| tumorigenic TD Mouse Oral | 69 | 114 | 0.61±0.09 | 0.92±0.04 | 0.95±0.07 | FXR-BLA agonist viability | 0.60 |
| tumorigenic TD Rat Oral | 74 | 139 | 0.52±0.09 | 0.77±0.05 | 0.59±0.21 | PPAR-gamma agonist ch1 | 0.57 |
| tumorigenic TDLo Mouse Injection subcutaneous | 63 | 121 | 0.7±0.1 | 0.72±0.07 | 0.69±0.12 | PPAR-gamma antagonist ch1 | 0.71 |
| tumorigenic TDLo Mouse Oral | 174 | 304 | 0.58±0.04 | 0.75±0.04 | 0.92±0.05 | GR-BLA antagonist viability | 0.60 |
| tumorigenic TDLo Mouse Skin | 52 | 99 | 0.69±0.1 | 0.86±0.04 | 0.83±0.19 | GR-BLA agonist ch2 | 0.65 |
| tumorigenic TDLo Rat Oral | 186 | 350 | 0.55±0.05 | 0.67±0.03 | 0.93±0.04 | GR-BLA antagonist viability | 0.55 |
| acute toxicity | 3532 | 2185 | 0.57±0.01 | 0.73±0.01 | 0.72±0.01 | DT40 Rev3 | 0.59 |
| reproductive | 860 | 1535 | 0.62±0.01 | 0.7±0.01 | 0.78±0.02 | DT40 WT | 0.63 |
| tumorigenic | 367 | 622 | 0.56±0.03 | 0.69±0.02 | 0.83±0.04 | TR-beta antagonist | 0.59 |
